# THE CEREBELLUM MAY MITIGATE OBESITY-DRIVEN COGNITIVE IMPAIRMENT IN LATE LIFE

**DOI:** 10.1093/geroni/igz038.358

**Published:** 2019-11-08

**Authors:** Valentina R Garbarino, Eric Baeuerle, Miranda E Orr

**Affiliations:** 1 UTHSA, San Antonio, Texas, United States; 2 Barshop Institute for Longevity and Aging Studies, UT Health San Antonio, San Antonio, Texas, United States; 3 GRECC, South Texas Veterans Health Care System, San Antonio, Texas, United States

## Abstract

Mice that overexpress mutant human tau in forebrain neurons develop many features of Alzheimer’s disease (AD), including behavioral impairments and neurodegeneration by 5 months of age. While an appropriate model to study AD-like pathology, the transgene’s high neurotoxicity makes it difficult to investigate how aging impacts AD onset and progression. The removal of endogenous mouse tau decreases the transgene’s neurotoxicity in young mice, which has allowed us to age mice to 20 months of age and investigate behavior at a more AD-relevant stage of life. Interestingly, the tau transgenic mice show increased discrimination between familiar and unfamiliar objects than non-transgenic littermates (p = 0.02) suggesting tau transgenic mice have better memory. The transgenic mice also displayed increased physical activity in the Open Field Test than non-transgenic littermates (distance traveled, p = 0.0102, and gait speed, p = 0.0219). Their improved behavioral performance occurred despite significant forebrain atrophy (20% smaller, p=0.0003). Interestingly, the non-transgenic control mice lacking endogenous mouse tau developed insulin resistance and obesity, and had significantly smaller cerebellum than transgenic mice (10% smaller, p = 0.0007). These data suggest that insulin resistance and obesity contribute more profoundly to poor behavioral performance than forebrain neurodegeneration. Moreover our study suggests that the cerebellum, recognized primarily for its role in coordination and motor function, may be an important mediator of late life cognitive function, especially in the presence of insulin resistance and obesity.

